# Desiccation Cracking Behavior of Sustainable and Environmentally Friendly Reinforced Cohesive Soils

**DOI:** 10.3390/polym14071318

**Published:** 2022-03-24

**Authors:** Michael Z. Izzo, Marta Miletić

**Affiliations:** 1 Department of Civil and Environmental Engineering, Auburn University, Auburn, AL 36849, USA; mzi0006@auburn.edu; 2 Department of Civil, Construction, and Environmental Engineering, San Diego State University, San Diego, CA 92182, USA

**Keywords:** desiccation cracking, biopolymers, cohesive soils, image processing, hydro-mechanical model, green ground improvement engineering

## Abstract

Desiccation cracking of cohesive soils is the development of cracks on the soil surface as a result of a reduction in water content. The formation of desiccation cracks on the cohesive soil surface has an undesirable impact on the mechanical, hydrological, and physicochemical soil properties. Therefore, the main aim of this study is to experimentally and numerically investigate eco-friendly soil improvement additives and their effect on the desiccation cracking behavior of soils. Improvement of soil crack resistance was experimentally studied by conducting desiccation cracking tests on kaolin clay. Biopolymer xanthan gum and recycled carpet fibers were studied as potential sustainable soil improvement additives. In addition, image processing was conducted to describe the effect of an additive on the geometrical characteristics of crack patterns. The results show that the soil improvement additives generally enhanced the soil strength and reduced cracking. Furthermore, a hydro-mechanical model was developed to predict the moisture transfer and onset of desiccation cracks in plain and amended kaolin clays. Data obtained show that the inception of the desiccation cracking and radial displacements were delayed in the improved soil specimens, which is in agreement with the experimental data.

## 1. Introduction

### 1.1. Background

Desiccation cracking is the development of cracks at the soil surface and throughout the depth of a cohesive soil layer as a result of moisture content loss. The desiccation of cohesive soils leads to an undesirable impact on the mechanical, hydrological, thermal, and physio-chemical soil properties. For instance, desiccation cracking can lead to decreased soil strength, which in turn can cause uneven soil settlement and catastrophic failures in structures of all types. The increase in soil permeability is particularly problematic when the clay is used as a liner for both landfills and hazardous waste storage [1], resulting in undesired paths for leachate. Increased permeability can also have a dramatic agricultural impact where water filters through soil far too quickly, vacating the root zone of vegetation before the required amount of water is absorbed. Therefore, the prevention of desiccation cracking is of significant importance to safety, structural, agricultural, and environmental issues.

In the most widely accepted explanation, desiccation cracks are formed as a result of soil volume shrinkage due to decreased water content, generally coming in the form of evaporation [2]. The shrinkage creates tensile stress within the soil and cracks develop when the tensile stresses experienced exceed the tensile strength of the soil. However, desiccation crack formation is a complicated process due to the interplay between the hydraulic and mechanical behavior of soils. Water loss during evaporation induces an increase in capillary forces, and the water transfer process in drying soils is controlled by the hydraulic properties of the soil mass. This, in turn, affects the mechanical behavior, because the soil tends to contract under increasing suction [3]. It has been stated that desiccation cracking is a coupled suction–contraction process. Suction and compressibility have been found to increase with the specific surface of the soil particles. Because clay particles are known to have the greatest specific surface of all soil particles, suction increases with clay content [4]. The increase in suction due to the water loss decreases the volume of the voids in the soil mass, introducing tensile stress.

Another view on this process is that shrinkage strain is a function of water content loss [5,6]. As the water content in cohesive soils decreases, the shrinkage strain increases. The shrinkage strain increase will cause the soil to shrink while being restricted by boundary conditions and material interfaces [6]. The restrictions on the shrinkage strain will cause the tensile stresses in the soil to increase, leading to desiccation cracking when the tensile strength of the soil is exceeded by the tensile stresses in any horizontal direction.

Crack initiation is also affected by the state of the soil surface [5]. Flaws in the soil surface can cause cracks to occur at locations other than the location of the maximum tensile stress. Fracture mechanics dictate that the tensile stress required to initiate a flaw is inversely proportional to the flaw size. Therefore, a flaw large enough positioned away from the location of maximum stress could still be the location of the initial crack.

### 1.2. Soil-strengthening Additives

To prevent desiccation cracking, several research studies have been conducted in order to determine the viability of different sustainable soil-strengthening additives. Soil additives can be considered sustainable if they have originated and have been refined from industrial waste, which remains toxic and hazardous to the environment when left unattended [7]. While this repurposing does not eliminate all negative side effects of the material as a waste product, it does prevent the adverse impacts of the use of alternative solutions, such as cement and lime [7,8]. The most commonly used sustainable soil additives are fly ash from thermal power plants [7,9,10], furnace slag from the steel industry [11,12], silica fume from the silicon and ferrosilicon industry [13,14,15,16], and carpet waste [17,18,19].

Different types of fibers have also been examined as potential solutions to desiccation cracking [13,14,20]. Recycled carpet fibers have been lightly investigated as a potential soil improvement technique, with most studies being focused on the improvement of the strength properties of granular soil [18,19]. More recently, they have been examined as a potential method to reduce the swelling properties of cohesive soils [17]. It should be noted that the distribution of fibers is important to their effectiveness in strengthening soils [13]. If homogeneity is not maintained, cracks can initiate along the paths of least resistance. Shorter fibers have been shown to be more effective in establishing a uniform fiber distribution, while longer fibers are more likely to bunch, diminishing the effect of adding the fibers.

Recycled carpet fibers [21] were shown to decrease the swelling pressure, with a fiber content of 1% providing the most significant pressure drop [17]. Other unsustainable fiber reinforcements have been shown to cause a substantial reduction in desiccation cracking in expanding clay, with the crack width decreasing by 50% [20]. Polypropylene fiber reinforcement has been proven to prevent tension crack growth in soils and stabilize soils against shrinking by increasing the soil’s tensile strength [14]. In addition, polypropylene fibers have been utilized to reinforce clay liners, leading the liners to be more rigid in compression and more ductile in tension [9]. Soils treated with fibers have also been shown to perform better than unreinforced soils when exposed to wetting and drying cycles [20].

In recent years, biopolymers have been examined as an avenue for the strengthening of soils [22,23,24,25,26]. Xanthan gum has been shown to improve the shear strength and compressive strength of soil [22,23,25,26]. The increased strength is due to the improved bonding that occurs between particles as a result of the presence of the xanthan gum [22,24,25]. Along with xanthan gum, guar gum, beta 1,3/1,6 glucan, chitosan, and alginate have been compared in a study where the benefits of each were determined for four mechanical strength tests [24]. Xanthan gum, guar gum, and beta 1,3/1,6 glucan were all shown to be effective in increasing the strength of soil [24].

### 1.3. Analytical and Numerical Modeling

Numerical models have been used as predictive tools for cracking in several different types of media. One of the approaches is the extended finite element method (XFEM) [27]. The XFEM has been utilized on a variety of different research topics, including reinforced concrete cracking [27], thermal reflective cracking [28], and soil slope stability [29]. Another finite element approach is the mesh fragmentation approach [30]. This method was developed primarily to tackle the problem of desiccation cracking. Using this approach, crack geometry, as well as the stresses and strains experienced by the soil, can be evaluated. A different modeling approach that still utilizes finite element software is the cohesive crack or cohesive segments approach [31]. This method inserts cohesive segments into finite elements, but only when the cohesive segments are necessary.

Other modeling approaches have also been employed to handle cracking behavior. The distinct element method has been used to model cracking behavior in various materials, often with the Universal Distinct Element Code (UDEC) software, which creates a continuum of finite difference elements [32,33]. The strength of distinct element programs lies in their ability to model the breaking up of material. The discrete element method (DEM) has proven to be another alternative way to model desiccation cracking [34,35,36,37]. With DEM models, discrete particles are used to replicate soils on the aggregate scale. While this works well on smaller-scale models, the computational power required to tackle larger problems provides a limit on its current viability [34].

Analytical models have also been used as predictive tools for cracking in soil [5,6,38,39]. For 1D desiccation tests, analytical models were capable of predicting not only initial cracking behavior but also additional cracking that occurred after [6]. The model [6] treated the clay layer as an elastic material, and shrinkage strains were applied to the clay as the soil dried. The longitudinal tensile stresses were then calculated from strains, with the maximum tensile stress occurring at the center of the specimen due to the boundary conditions associated with 1D desiccation tests. When the tensile stress exceeded the tensile strength of the soil, a crack would occur. After the crack initiated, the model would be split into essentially two specimens, and the process is repeated until cracking transpires again. The recursive process allows for crack spacing to be examined at the end of total crack propagation.

One desiccation theory of soft, fine-grained soils creates a model that considers the growth of vertical cracks and 1D desiccation under 3D shrinkage [38]. The key aspect of this model is the examination of the effective stress path of a soil layer while undergoing consolidation. Much like many other crack initiation determinations, cracking occurs when tensile stress surpasses the tensile strength of the media. A different model utilizes a similar approach yet provides a method for predicting the average spacing between primary cracks in conjunction with crack initiation and crack depth [39]. Linear elastic fracture mechanics and a stress superposition concept are used to add crack spacing as a prediction parameter. Initial cracking occurs when the tensile stress is greater than the tensile strength of the soil. Crack depth is then determined using a trapezoidal stress distribution along with linear elastic fracture mechanics and an effective stress path that is dependent on the soil type. Finally, a horizontal stress relief distribution is utilized to determine crack spacing with neighboring cracks existing when 95% of the tensile strength of the soil layer is exceeded. This model was compared with field observations for China clay with promising results.

While previous models examined 1D cracking, a 2D analytical model is also possible [5]. A polar coordinate system was adopted to handle the circular specimen shape of many desiccation tests. Using the change in water content as desiccation occurs, variable material properties, such as Young’s modulus and tensile strength, and potential shrinkage strain were determined. Stresses were calculated on the basis of two boundary conditions. The first boundary condition was satisfied while the soil remained adhered to the mold wall. The second boundary condition was satisfied after the soil had detached from the mold wall. While this model examines 2D horizontal cracking, it cannot predict crack spacing or crack depth.

### 1.4. Scope of Research

This study aims to investigate novel eco-friendly soil improvement techniques and their effect on the desiccation cracking behavior of soils. The type of cohesive soil that was used as part of this investigation was pure white kaolin clay. The two sustainable soil improvement additives were recycled carpet fibers and biopolymer xanthan gum. Recycled carpet fibers were considered as sustainable materials under the idea that repurposed industrial waste is a sustainable material. However, their effect on the crack resistance of cohesive soils has been largely under-investigated. A comparative analysis of the mentioned improvement additives’ effectiveness has not been completed.

A qualitative analysis was conducted by analyzing the geometric characteristics of cracking patterns in the plain and improved soil samples. The quantitative analysis was completed using the image analysis software GOM Correlate. A comparative analysis of the soil improvement additives was completed using a combination of the maximum crack width (*w_max_*), maximum crack length (*l_max_*), and maximum radial shrinkage (*rs_max_*) of the treated and non-treated cohesive soil samples.

To corroborate the results from the physical tests, a combined numerical–analytical model was developed with the goal of projecting the cracking behavior of cohesive soils amended with different soil improvement additives. The model created a field of water contents across both the depth of the soil layer and time. These water contents were then used to determine the shrinkage strain and the stress conditions with changes in the time, depth, and radial distance. Two stress conditions were considered, where the radial and hoop stresses under a polar coordinate system were calculated based on whether the soil was still attached to the mold. The stresses were then used to predict the time of crack initiation as well as the radial displacement of the clay specimens for each improvement technique. The results of the hydro-mechanical model were then compared to the experimental results as a way of evaluating and validating the model.

## 2. Materials and Experimental Methodologies

### 2.1. Base Soil and Additives

**Figure 1:**
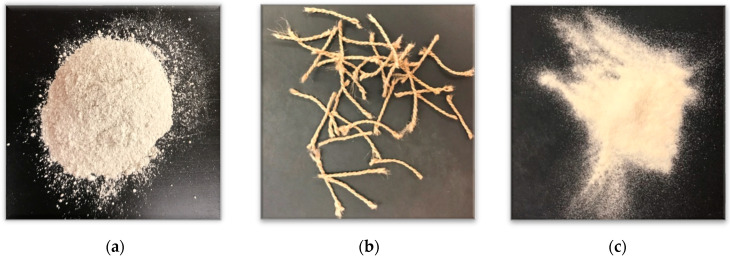
Photos of (**a**) dry kaolinite clay; (**b**) recycled carpet fibers; and (**c**) xanthan gum.

The soil used in this study was white kaolin clay. The soil was tested for its Atterberg Limits in accordance with ASTM D4318 [40], and the Liquid Limit and Plasticity Index were determined to be 45 and 13, respectively. Therefore, according to the Unified Soil Classification System, the kaolin clay utilized in the study was classified as lean clay. The specific gravity of the clay was 2.4 [41]. A photo of the dry kaolinite clay can be seen in Figure 1a.

The additives investigated were recycled polyester (poly(ethylene terephthalate)) carpet fibers at two different volumetric fiber contents and biopolymer xanthan gum. The polyester carpet fibers were short fibers manufactured by Beaulieu of America, Dalton, GA, USA. They had a length of 10 mm with a consistent diameter of 0.3 mm (an aspect ratio of 33). The density of the polyester fibers was 1.38 g/cm^3^ (20 °C), and the volumetric fiber contents utilized included 0.5% and 2%. A photo of the isolated recycled carpet fibers can be found in Figure 1b.

The biopolymer xanthan gum is produced through the fermentation of glucose and sucrose [24]. Molecularly, xanthan gum is a polysaccharide or a long chain of carbohydrate molecules. Like many other polysaccharides, xanthan gum has been used as a thickening agent for a variety of substances and to increase the viscosity of liquids. In this study, samples with a mass concentration (the biopolymer mass ratio to the soil mass) of 1% xanthan gum were tested. A photo of the isolated dry xanthan gum can be found in Figure 1c.

### 2.2. Desiccation Test

The desiccation test was performed on improved and non-improved kaolin clay specimens. The purpose of the desiccation test was to study the evolution and distribution of soil suction, water content, and cracking behavior of a soil sample as the sample reduces the moisture content and dehydrates. The soil samples were prepared by first mixing all the dry components (kaolin clay, additives) for a few minutes to ensure a uniform distribution. After mixing an additive with clay, water (up to 60% of the mass of the plain clay) was sprayed into the clay–additive mixture. Once thoroughly mixed, the soil specimens were manually placed into the cylindrically shaped, transparent plastic molds and lightly smoothed to a uniform thickness of 30 mm. The inner diameter of the mold was 190 mm. After the sample was thoroughly prepared, a random speckle pattern was applied on the surface of the clay samples to enhance the image analysis.

**Figure 2:**
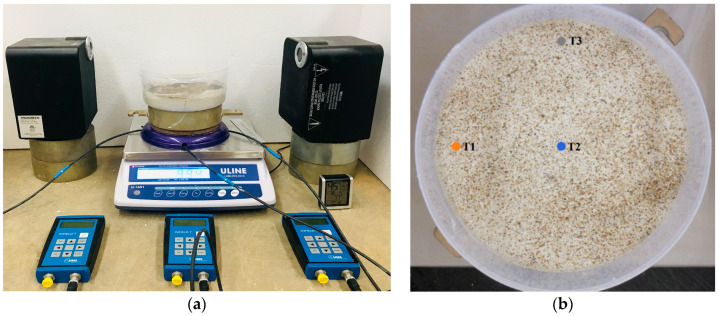
Photos of (**a**) the experimental setup for the desiccation test, and (**b**) tensiometer locations (T1, T2, and T3).

The plain soil and each of the additive configurations were tested under same conditions. All the tests were performed at a constant relative humidity and temperature (40% and 30 °C, respectively). For each test, three samples were used. The soil suction, moisture content, sample height, and development of desiccation cracking were continuously monitored and recorded over the duration of the experiment (six hours). The entire experimental setup can be seen in Figure 2a.

The moisture content of the specimen was recorded with respect to time by continually weighing a sample. METER UMS Miniature-Tensiometer T5 tensiometers were used to measure soil suction. These tensiometers were capable of recording suctions in the range 100 to −85 kPa, which can be performed in five seconds or less. The tensiometers were placed in three consistent locations. A schematic of the tensiometer alignment can be seen in Figure 2b.

### 2.3. Digital Image Acquisition and Processing

Digital images of the soil samples were taken at fixed time increments using a stand held at a constant position for image consistency and uniformity. A Canon Powershot ELPH 360 HS digital camera was set up on a tripod to provide an aerial view of the crack initiation and propagation throughout the test. The tripod was set up at a constant height of 900 mm for each test. Digital photos were recorded every thirty minutes.

**Figure 3:**
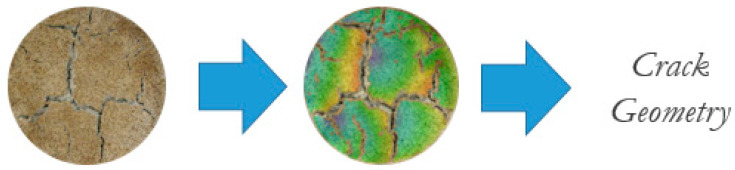
Image processing using GOM Correlate.

The digital image acquisition was followed by image processing to conduct a quantitative analysis of the crack geometric properties within the soil sample. To do so, digital image analysis was completed for each of the specimens that were tested in the desiccation experiment. The series of photos corresponding to each data point (Figure 3—left) were uploaded in chronological order to the image processing software GOM Correlate [42]. GOM Correlate functions by reading a dense speckle pattern on a series of photos. The program tracks the displacement of the speckles as time passes by a chronological digital still.

Before tracking the displacement, the photos were calibrated by measuring an element of known length and applying that distance to all the photos in the sequence. Afterward, a surface component was set, which essentially selects the region of importance in all of the images. For this procedure, this means choosing the clay surface. When configured correctly, the only discontinuities in the component would be where cracking occurs. At this point, the overall displacement was mapped on the images as a heat map on each image (Figure 3—center). The location with the greatest displacement away from the component edge was used to identify the maximum crack width. The maximum crack width was then determined by locating the longest continuous crack path on each image and measuring it with the measuring tool. Similar to the maximum crack width, the radial shrinkage was determined by using the heat map. However, the radial shrinkage was viewed as an average value, and the average of the values found on the edge of the surface component was recorded. The general process for the GOM Correlate image analysis can be seen in Figure 3.

## 3. Hydro-Mechanical Model

### 3.1. Soil Water Flow Equations

The movement of water in unsaturated soils can be represented with Richard’s equation [43,44,45]. It is a nonlinear partial differential equation that is often challenging to approximate since it does not have a closed-form analytical solution. The main objective of the hydraulic modeling process was to develop a numerical solution to the head-based form of Richard’s equation, which can be seen in Equation (1):

(1) $$C\left(h\right)\frac{\partial h}{\partial t}=\frac{\partial }{\partial z}\left[K\left(h\right)\frac{\partial h}{\partial z}\right]-\frac{\partial K\left(h\right)}{\partial z},\;0\;\leq \;z\;<\;L;\;\;t\;>\;0$$

where *h* is the pressure head, *t* is the time, *z* is the depth, *K*(*h*) is the unsaturated hydraulic conductivity, and *L* is the soil layer depth. *C*(*h*) is a function describing the rate of change of saturation with respect to the pressure head and is defined as follows:

(2) $$C\left(h\right)=\frac{d\theta }{dh}$$

where *θ* is the volumetric water content.

The van Genuchten [46] model, which defines the hydraulic conductivity as a function of the pressure head,

(3) $$K\left(h\right)=K_{s}\frac{{\left[1-{|\alpha h|}^{n_{G}-1}{\left(1+{|\alpha h|}^{n_{G}}\right)}^{-m_{G}}\right]}^{2}}{{\left(1+{|\alpha h|}^{n_{G}}\right)}^{\frac{m_{G}}{2}}}$$

and describes the soil water retention curve (SWRC):

(4) $$\theta =\frac{\theta_{s}-\theta_{r}}{{\left(1+{|\alpha h|}^{n_{G}}\right)}^{m_{G}}}+\theta_{r}$$

was used in this study. In the above equations, *Ks* is the saturated hydraulic conductivity, α is an empirical constant with units in cm^−1^, *θs* is the saturated water content, and *θr* is the residual water content. The parameters *m_G_* and *n_G_* are dimensionless empirical constants where the relationship between the two must be defined by:

(5) $$m_{G}=1-\frac{1}{n_{G}}$$

The specific water capacity can then be defined by differentiating Equation (3) to yield:

(6) $$C\left(h\right)=\frac{n_{G}m_{G}\alpha \left(\theta_{s}-\theta_{r}\right)}{{\left(1+{|\alpha h|}^{n_{G}}\right)}^{m_{G}+1}}{|\alpha h|}^{n_{G}-1}$$

Because the desiccation phenomena can be simplified as the vertical evaporation of water from soil with an initially uniform pressure head and a constant flux at the surface throughout the time of analysis, the initial and boundary conditions are defined as follows:

(7) $$q\left(0,t\right)={\left[-K\left(h\right)\frac{dh}{dz}+K\left(h\right)\right]}_{z=0}=q_{a};t>0$$

and

(8) $${\left[\frac{\partial h}{\partial z}\right]}_{z=L}=0;t>0$$

and

(9) $$h\left(z,0\right)=h_{b};0<z<L$$

where *h_b_* is the initial uniform pressure head and *q_a_* is the constant evaporation flux. Ultimately, the Crank–Nicholson finite-difference method [47] was selected as an approximation approach to derive the water content as a function of time, pressure head, and depth.

### 3.2. Hydro-Mechanical Coupling

To fully model the desiccation cracking process, the hydraulic and mechanical behavior need to be coupled. To couple moisture transfer and soil deformation, the one-way coupling was used, and it was achieved through the use of the hydric constant α_w_ [5,6]. The hydric constant assumes a linear relationship between gravimetric water content loss (positive during drying) and shrinkage strain $\epsilon_{sh}$ as a percentage, which can be seen in the following equation:

(10) $$\epsilon_{sh}=\alpha_{w}\Delta \theta$$

For each time increment, the hydraulic analysis was completed as described above, and the output corresponding to the water content values was converted into shrinkage strains and utilized as an input in the mechanical problem. This results in displacement and stresses that are different from those obtained by simulating dry conditions. However, the hydrological parameters are assumed to be independent of strain. The corresponding results from the mechanical model are not input into the hydraulic model; therefore, model cracking behavior is only valid until the time of initial crack formation.

### 3.3. Mechanical Behavior

In order to complete the hydro-mechanical model, the following analytical solution was implemented for desiccation cracking in circular soil layers [5]. During the desiccation and soil moisture evaporation, material properties and mechanical boundary effects will change [5,6]. Up to the onset of desiccation cracking, the soil was assumed to be a macroscopically homogenous elastic continuum exhibiting axisymmetry. In this study, desiccation crack initiation was based on the simple tensile failure criterion of the tensile stress exceeding the material’s tensile strength. All stresses in the vertical direction due to the self-weight were neglected. In addition, the soil layer was assumed to be in the plane-stress condition, because it is thin and is subjected to uniform stress with a stress-free surface at the top.

After resolving the stresses and forces acting on the infinitesimal, axisymmetric element, the following equation of stress equilibrium can be obtained:

(11) $$\left(\sigma_{r}+d\sigma_{r}\right)\left(r+dr\right)Hd\Theta -\sigma_{r}\left(rd\Theta \right)H+\tau rd\Theta dr\;-\frac{\sigma_{\Theta }d\Theta }{2}Hdr-\frac{\left(\sigma_{\Theta }+d\sigma_{\Theta }\right)d\Theta }{2}Hdr=0$$

where *σ_r_* is the radial shrinkage stress; *σ*_*Θ*_ is the hoop shrinkage stress; *r* is the length along the radius of the clay specimen, from the center out; *H* is the specimen thickness; *τ* is the shear strength at the base of the sample due to the soil–mold interface; *Θ* is the angle in from a reference direction in the polar coordinate system; and *dΘ* is the angle differential.

Through rearranging terms in Equation (11), the following differential equations of stress equilibrium can be achieved:

(12) $$\frac{d\sigma_{r}}{dr}+\frac{\sigma_{r}-\sigma_{\Theta }}{r}=-\frac{\tau }{H}$$

The water content has a significant effect on the soil’s mechanical behavior and properties. Similarly to the theoretical treatment of thermal stresses in solids [48], some authors have suggested that the shrinkage in soil layers can be proportional to the change in moisture content [5,6,49]. In this approach, soil suction is not required for the stress–strain constitutive equations, which is an advantage due to the difficulty and complexity of experimentally measuring soil suction. In contrast, the evolution of water content with time can be obtained easily in practical engineering. However, if needed, the material properties can still depend on the soil suction as the desiccation progresses.

Using elasticity theory, the radial and hoop stresses can be defined as:

(13) $$\sigma_{r}=\frac{E}{1-v^{2}}\left(\epsilon_{r}^{T}+v\epsilon_{\Theta }^{T}\right)$$

(14) $$\sigma_{\Theta }=\frac{E}{1-v^{2}}\left(\epsilon_{\Theta }^{T}+v\epsilon_{r}^{T}\right)$$

where *E* is Young’s Modulus, *v* is Poisson’s ratio, $\epsilon_{r}^{T}$ is the total radial strain, and $\epsilon_{\Theta }^{T}$ is the total strain in the circumferential direction.

The total radial and total circumferential strains are defined as the sum of the actual (or mechanical) strain ($\epsilon_{r}$ and $\epsilon_{\Theta }$) and the shrinkage strain ($\epsilon_{sh})$:

(15) $$\epsilon_{r}^{T}=\epsilon_{r}+\epsilon_{sh}$$

(16) $$\epsilon_{\Theta }^{T}=\epsilon_{\Theta }+\epsilon_{sh}$$

where $\epsilon_{sh}$ is the shrinkage strain defined by the model coupling. Furthermore, $\epsilon_{r}$ and $\epsilon_{\Theta }$ are the actual radial and circumferential strains, respectively, given as:

(17) $$\epsilon_{r}=\frac{dU_{r}}{dr}$$

(18) $$\epsilon_{\Theta }=\frac{U_{r}}{r}$$

where *U_r_* is the displacement in the radial direction.

Substituting Equations (15) through (18) into Equations (13) and (14) yields the following expressions for the radial and hoop stresses:

(19) $$\sigma_{r}=\frac{E}{1-v^{2}}\left[\frac{dU_{r}}{dr}+v\frac{U_{r}}{r}+\left(1+v\right)\epsilon_{sh}\right]$$

(20) $$\sigma_{\Theta }=\frac{E}{1-v^{2}}\left[\frac{U_{r}}{r}+v\frac{dU_{r}}{dr}+\left(1+v\right)\epsilon_{sh}\right]$$

Furthermore, substituting Equations (13) through (18) into Equation (12) gives:

(21) $$\frac{d^{2}U_{r}}{dr^{2}}+\frac{1}{r}\frac{dU_{r}}{dr}-\left(1+v\right)\frac{d\epsilon_{sh}}{dr}=-\frac{\tau \left(1-v^{2}\right)}{EH}$$

Because of the assumed isotropic conditions in the clay specimen, $\frac{d\epsilon_{sh}}{dr}$ can be assumed to be zero. Therefore, Equation (21) reduces to the equation below:

(22) $$\frac{d}{dr}\;\left[\frac{1}{r}\frac{d\left(rU_{r}\right)}{dr}\right]=-\frac{\tau \left(1-v^{2}\right)}{EH}$$

Integrating Equation (22) and then differentiating the result yields Equations (23) and (24), respectively:

(23) $$U_{r}=-=-\frac{\tau \left(1-v^{2}\right)}{EH}\frac{r^{2}}{3}+\frac{Ar}{2}+\frac{B}{2}$$

(24) $$\frac{dU_{r}}{dr}=-=-\frac{\tau \left(1-v^{2}\right)}{EH}\frac{2r}{3}+\frac{A}{2}$$

In the above equations, both *A* and *B* are integration constants. From here, two mechanical boundary condition scenarios are analyzed.

#### 3.3.1. Pre-Wall Crack Stress Derivation

The first scenario is when the clay specimen is still attached to the vertical wall of the mold. At this point, the radial stress is less than the interface adhesion between the sample and the mold. Therefore, in this circumstance, *U_r_* is equal to zero when *r* = 0 and when *r* is equal to the maximum radial distance (*r*_0_). Because no radial displacement occurs in this environment, *τ* can be assumed to be zero as well. With the above information, Equations (23) and (24) are solved for constants A and B and plugged into Equations (19) and (20) to obtain the radial and hoop stresses for the attached wall scenario:

(25) $$\sigma_{r}=\frac{E}{1-v}\epsilon_{sh}$$

(26) $$\sigma_{\Theta }=\frac{E}{1-v}\epsilon_{sh}$$

#### 3.3.2. Post-Wall Crack Stress Derivation

Once the radial stress evaluated under the previous conditions exceeds the adhesion value between the mold and clay, the second scenario is evaluated. In this environment, the specimen edge is free to be displaced in the negative radial direction. This yields new boundary conditions where *U_r_ = 0* when *r = 0* and *σ_r_ = 0* when *r = r_0_.* With these new boundary conditions, Equations (23) and (24) are solved for constants A and B and plugged into Equations (19) and (20) to obtain the radial stress, hoop stress, and radial displacement for the free movement scenario:

(27) $$\sigma_{r}=\frac{\tau \left(2+v\right)}{3H}\left(r_{0}-r\right)$$

(28) $$\sigma_{\Theta }=\frac{\tau }{3H}\left[\left(2+v\right)r_{0}-\left(1+v\right)r\right]$$

(29) $$U_{r}=\frac{\tau \left(1-v\right)r}{3EH}\left[\left(2+v\right)r_{0}-\left(1+v\right)r\right]-r\epsilon_{sh}$$

#### 3.3.3. Stress State Discussion

The equations above provide a reliable avenue for analysis of the stress field under both sets of boundary conditions. Equations (25) and (26) show that the radial stress and hoop stress are equal at all points, meaning a uniform tensile stress field exists under ideal conditions. This creates a situation where the first crack will most likely occur along the soil–mold interface as the tensile strength of the soil is generally higher than the adhesion of soil to the mold. In the rarer scenario where the tensile strength of the soil is less than the adhesion value, a crack would be equally likely to occur anywhere in the soil surface.

Once the wall crack has formed, the new stress and displacement distributions are based on Equations (27)–(29). At this point, the radial stress and the hoop stress possess an inverse linear correlation along the radius with the maximum stress occurring at the center of the specimen. However, they do not decrease at the same rate. Whereas the radial stress will decrease to zero at the edge of the specimen, the hoop stress will maintain a residual value at the edge. As a result, the hoop stress is greater than the radial stress at all points in the specimen, aside from the center. This results in cracks initiating at the center and radiating out to the edge, perpendicular to the greater hoop stress. Real-life crack patterns, however, will diverge from this pattern and will be governed by flaws in the soil surface, specifically their size and orientation [5].

### 3.4. Material Properties and Input Parameters

In order to employ the combined analytical–numerical hydro-mechanical model detailed above, several kaolin clay material properties and environmental factors were required. The following is a description of how these values were obtained for the untreated soil and each improvement technique.

The van Genuchten empirical constants (Equation (4)) were determined to be constant for all soil improvement techniques. The saturated volumetric water content, $\theta_{s}$, was determined to be 0.77 from the initial water content in the physical desiccation tests. The residual volumetric water content, $\theta_{r}$, was determined by experimental data on kaolin soils [43,44] and was found to be zero. The remaining parameters were determined by taking the SWRC returned from the physical experiments and fitting the van Genuchten water content equation to the data (Equation (4)). This yielded a value for *α* of 0.008478 cm^−1^, *n_G_* being 1.4228, and a corresponding *m_G_* of 0.29716.

The soil surface evaporation flux was assumed to be constant based on the linear water content loss during the desiccation tests. This returned a value of 0.0925 cm/hr. The hydric constant was the ratio of radial shrinkage strain to water content loss and was determined by calculating the slope of the experimental data of the radial shrinkage strain and water content loss relationship. Because the xanthan gum sample did not separate from the mold wall, a hydric constant for xanthan gum could not be determined and was assumed to be the same as the untreated soil. The hydric constant for untreated soil was found to be 0.1774. The hydric constants for the 0.5% and the 2.0% fiber-aided soils were found to be 0.1934 and 0.2665, respectively. For the Young’s modulus, tensile strength, interface shear strength, and interface adhesion, each value was considered to be a function of the water content based on experiments done in previous work on kaolin soil [50,51,52,53,54,55,56].

## 4. Results

### 4.1. Desiccation Test

**Figure 4:**
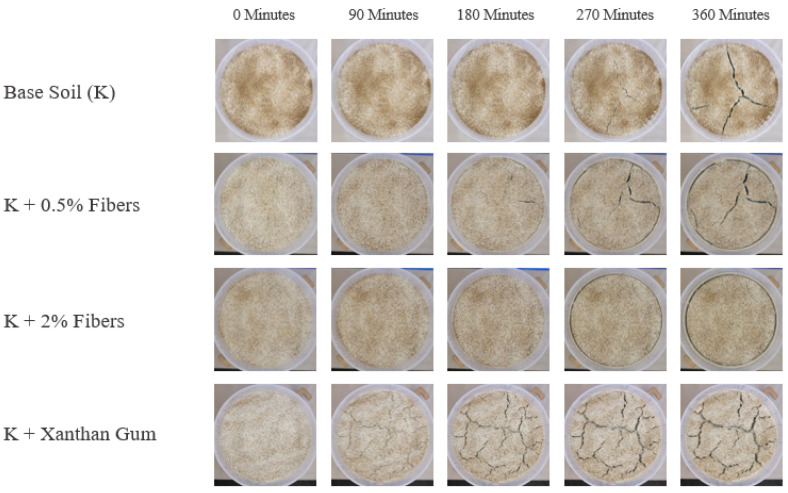
Desiccation test results during the six hours of drying.

Using the data obtained from the image analysis process, the performance of each of the selected improvement methods was studied. The *w_max_, l_max_*, and *rs_max_* were obtained for each time frame during the desiccation test. The progression of the cracking throughout the desiccation test can be seen in Figure 4.

Generally, the cracks appeared almost at the same time with the exception of the xanthan-gum-aided specimens, where crack initiation occurred much earlier. Each of the fiber-aided specimens first began to crack between the 90 and 180-min marks, quite similarly to the untreated kaolin clay. This suggests that the recycled fibers do not alter the crack initiation process but rather retard the propagation portion of the entire desiccation cracking phenomenon. Meanwhile, the samples treated with xanthan gum cracked immediately, with each sample cracking in the first 30-min heating interval, suggesting that the addition of the xanthan gum affected the crack formation mechanism of the clay. In general, the xanthan-gum-aided samples behaved in a much different fashion than each of the other kaolinite samples. Aside from cracking at an earlier time, the xanthan gum samples adhered to the sides of the mold to a much greater degree. In the authors’ opinion, this occurred due to the sides of the molds being more exposed to the heat source and the xanthan gum melting to a degree and behaving like a glue between the clay specimen and the mold.

**Figure 5:**
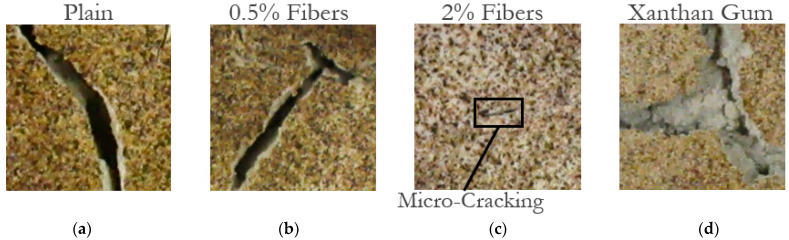
Comparison of cracks in kaolinite soil in (**a**) untreated samples, (**b**) samples aided with 0.5% fibers, (**c**) samples aided with 2% fibers, and (**d**) xanthan-gum-aided samples.

Furthermore, the cracks themselves developed to possess very different characteristics. Whereas the untreated kaolinite samples and the fiber-treated samples cracked in similar ways, albeit with differing magnitudes, the xanthan-gum-aided samples cracked in an altogether different manner. The pictures in Figure 5 contrast the difference between the cracks that formed. The general form of cracking in plain and fiber-aided clay can be seen in Figure 5a–c. Here, the cracks have clearly defined edges, with mostly vertical walls. In Figure 5d, the cracks for the xanthan gum samples can be observed. In these samples, the cracks seem to almost have a shallow shelf and a narrow deeper second phase. Additionally, the edges of these cracks are not nearly as well defined and seem to have almost crumbled to some degree.

**Figure 6:**
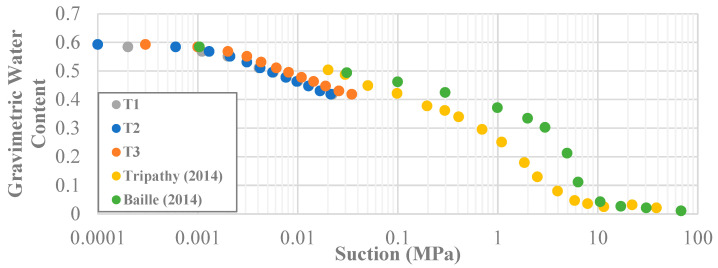
Soil Water Retention Curve for plain kaolinite. Other data are shown for comparison [43,44].

In addition, the water content and soil suction were recorded at each stage for every sample for the untreated soil and each improvement technique. The suction and gravimetric water content results were used to develop a SWRC (Figure 6). Because the water contents and suction levels were fairly identical throughout the testing process, only the results from the pure kaolinite clay were plotted for each position of the tensiometers. The SWRCs from other research projects on kaolinite clay were plotted as well [43,44].

**Figure 7:**
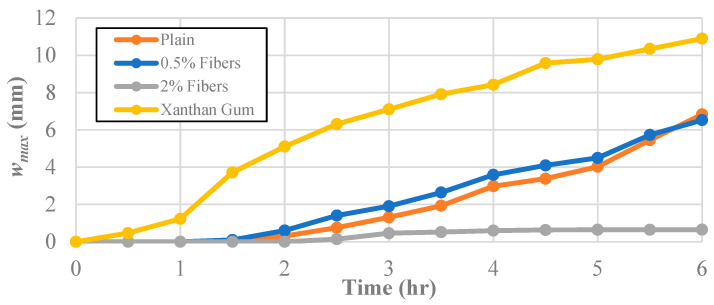
The effect of different additives on the maximum crack width, *w_max_.*

The effect of the tested soil improvement additives on the maximum crack width, *w_max_*, can be seen in Figure 7. Because three trials were completed, the average maximum crack width was utilized in order to minimize the effect of an outlying behavior. The maximum crack width at the end of the test for the untreated soil was 6.83 mm. The specimen treated with 0.5% fiber content developed a crack geometry with a slightly smaller maximum crack width than the plain kaolinite clay (6.53 mm). In other words, the progression between the untreated kaolinite and the samples treated with a fiber content of 0.5% was nearly identical, suggesting that the fiber content was not sufficient to alter the crack width behavior of the kaolinite significantly. Increasing the fiber content added to 2% had a much more significant effect as the maximum crack width achieved was only 0.65 mm. Crack growth in those samples effectively stopped after the four-hour mark, increasing by only 0.05 mm after that point. On the other hand, the samples treated with xanthan gum performed far worse than the untreated soil, with the cracks growing to a maximum width of 10.89 mm. In fact, at the three-hour mark, the maximum crack width exceeded the final maximum crack width for the other improvement techniques and the untreated clay.

**Figure 8:**
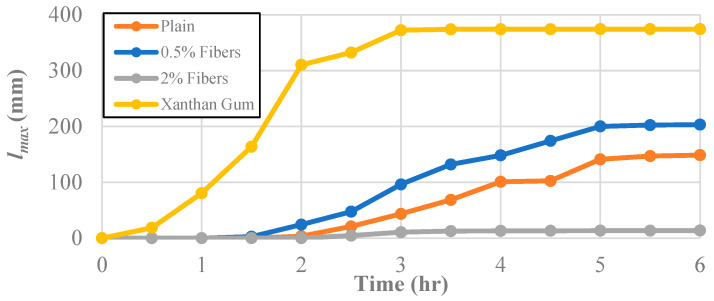
The effect of different additives on the maximum crack length, *l_max_.*

The effect of the tested soil improvement additives on the average maximum crack length, *l_max_*, can be seen in Figure 8. Initially, the maximum crack length was taken to be the most prolonged individual crack, but once the crack patterns became more complex, the longest continuous path in the crack network that did not intersect itself was used to define the *l_max_*. The reason for studying the maximum crack length in each specimen was to examine the desiccation crack network and each improvement technique’s aptitude for preventing the initiation of new cracks. The maximum crack length at the end of the desiccation test for the untreated soil was 149 mm.

The specimen treated with 0.5% fiber content developed a crack geometry with a slightly higher maximum crack length (203 mm) than the untreated clay. While the addition of the fibers did not dramatically change the crack width growth, the soils treated with the 0.5% fiber content promoted a more extensive crack network as seen in Figure 8.

Boosting the fiber content added to 2% had a more dramatic effect on the soil’s performance than the maximum crack length. The introduction of more fibers led to a decrease in the maximum crack length by a factor of nearly 15, i.e., to 13.6 mm. Again, crack growth in those samples essentially ended after the three-hour mark, increasing by approximately 5 mm after that point.

On the other end of the spectrum, the samples treated with xanthan gum performed perhaps even worse when considering the maximum crack length. With a maximum crack length of 374 mm, an increase of nearly 250% over the untreated soil, the samples treated with xanthan gum created a far more extensive crack network. Moreover, after two hours, the *l_max_* for the xanthan-gum-treated clay was more than double the final maximum crack length of the untreated clay.

**Figure 9:**
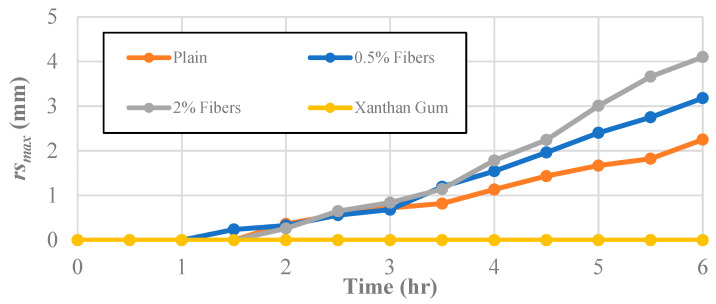
The effect of different additives on the maximum radial shrinkage, *rs_max_.*

In addition to the cracking characteristics, image processing was used to determine the effect of the different soil improvement techniques on the maximum radial shrinkage displacement, *rs_max_*, of the clay specimens (Figure 9). The impetus for studying the maximum radial shrinkage in each specimen was to contrast how the specimens reacted to a loss in water content between the global surface area loss and the cracking behavior.

The maximum radial shrinkage at the end of the desiccation test for the untreated soil was 2.25 mm. The radius length of the specimen treated with 0.5% fiber content decreased at a slightly higher rate with a final maximum radial shrinkage of 3.18 mm. This shows that the inclusion of a small number of fibers leads to a more extensive crack network and more significant radial shrinkage. While the addition of the fibers did not dramatically change the crack width growth, the soils treated with the 0.5% fiber content promoted a more extensive crack network.

Notably, utilizing the 2% fiber content method and the xanthan gum method yielded opposite results to the crack geometry results. The addition of the recycled fibers at a rate of 2% increased the radial shrinkage of the clay to 4.10 mm. These results, combined with the minimal crack propagation of the 2% fiber specimens, suggest that the fibers were able to increase the soil’s tensile strength to overcome the frictional stress on the bottom of the sample and the adhesion of the sample to the side of the molds, leading to global shrinkage instead of cracking.

Conversely, while the xanthan-gum-enhanced samples displayed the most expansive crack network, no radial shrinkage occurred within those specimens. Essentially, the specimens maintained contact with the plastic mold at all times and points. This suggests that the xanthan gum potentially increased the adhesion between the mold and the soil to the point where the soil cracked rather than moved away from the mold.

### 4.2. Hydro-Mechanical Model

#### 4.2.1. Hydraulic Model

**Figure 10:**
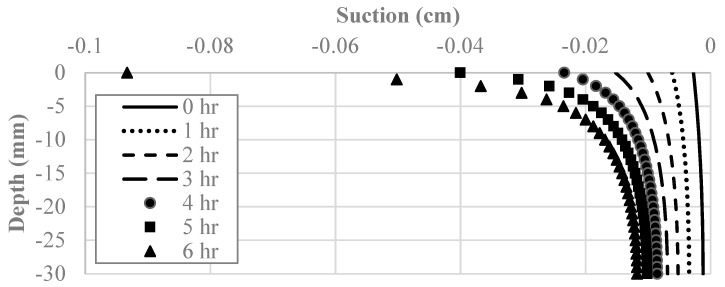
Suction as a function of depth and time in the hydro-mechanical model.

Figure 10 depicts the suction along with the depth of the soil layer, with the time step representing each 60-min increment shown. These curves are the same for each soil improvement technique because the additives did not affect moisture loss or the behavior of the SWCC. As can be seen in Figure 10, the magnitude of the suction experienced in the clay specimen increases with each time step and decreases as the depth increases. For instance, the suction at the soil surface changed by −9 kPa over six hours, while the change in suction at the bottom of the layer was only −0.96 kPa.

**Figure 11:**
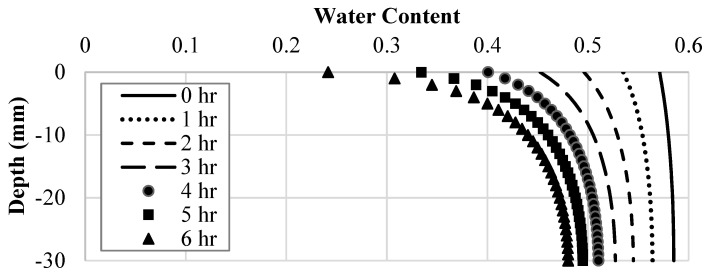
Water content as a function of depth and time in the hydro-mechanical model.

Creating a water content field across the depth of the clay layer for each time step was the most valuable result calculated by the hydraulic model due to water content being an input in the model coupling process. As such, the gravimetric water content along the depth of the soil layer was plotted, with the time step representing each 60-min increment shown. As can be seen in Figure 11, the magnitude of the water content existing in the clay specimen decreases with each time step and increases as the depth increases. The water content at the soil’s surface changed by −0.33 over six hours, while the change in water content at the bottom of the layer was only −0.1. The observed trend was expected as the water could only evaporate the clay specimen through the top soil surface, making the water content at that point the lowest at all times. This also reflects the suction behavior because unsaturated soil mechanics dictate that a decrease in water content increases the soil suction.

#### 4.2.2. Mechanical Model Results

**Figure 12:**
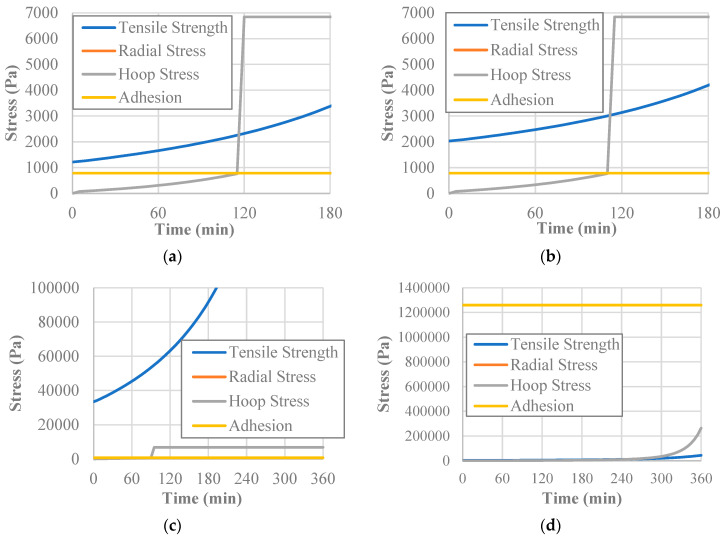
Stress progression at the center of the soil surface over time for the (**a**) untreated; (**b**) 0.5% fiber-treated; (**c**) 2% fiber-treated; and (**d**) xantham-gum-treated specimen.

After the hydraulic model was executed, the water content at each time and depth was input into the mechanical model for each soil improvement additive. Doing this allowed for the stresses at each point in the model’s profile to be calculated during the six-hour time period. The obtained data were then compared to the soil’s material and interface properties to determine the onset of desiccation cracking at different points in time, depths, and radial distances. Based on the stress formulation for the pre-wall crack scenario, the stresses produced at the center of the soil would always be equal to or greater than at any other point in the clay profile. As a result, this position in the soil layer was considered to be the critical one. Figure 12a shows how the stresses and governing material and interface properties progressed with time for the untreated soil at the center of the soil surface. At the center of the soil specimen, the radial stress and the hoop stress are identical and therefore overlaid in the figure.

Furthermore, it can be seen from Figure 12a that, at the 115-min mark, the pre-wall crack radial stress reaches the adhesion value. This means that the edge crack has formed, and therefore the specimen has detached from the wall. At this point, the radial and hoop stress calculations switch to the post-wall crack formulations, resulting in an immediate jump in the hoop and radial stress. This jump increases the stresses to such a degree that the tensile strength of the soil has also been surpassed, meaning that desiccation cracking will initiate.

The soil treated with 0.5% fiber content behaves in a similar way to the plain soil, with desiccation cracking behavior occurring 5 min earlier as shown in Figure 12b. This means that the increase in the hydric constant, and, subsequently, the shrinkage strain caused the stress to exceed the adhesion at an earlier stage. The soil treated with 2% fiber content deviates in its behavior when compared with the previous two examples as shown in Figure 12c. The added tensile strength from the addition of the recycled carpet fibers vastly overwhelms the jump when switching stress formulations. The behavior of the soil improved by xanthan gum strayed even farther from the behavior of the other samples (Figure 12d). Whereas all the other improvement methods broke their bond with the vertical wall of the mold, the increased adhesion led to the xanthan-gum-aided model never producing a wall crack. However, unlike the other specimens, the stresses from the pre-wall crack stress formulation exceeded the tensile strength toward the end of the time period, meaning that desiccation cracking will occur without separation from the mold.

**Figure 13:**
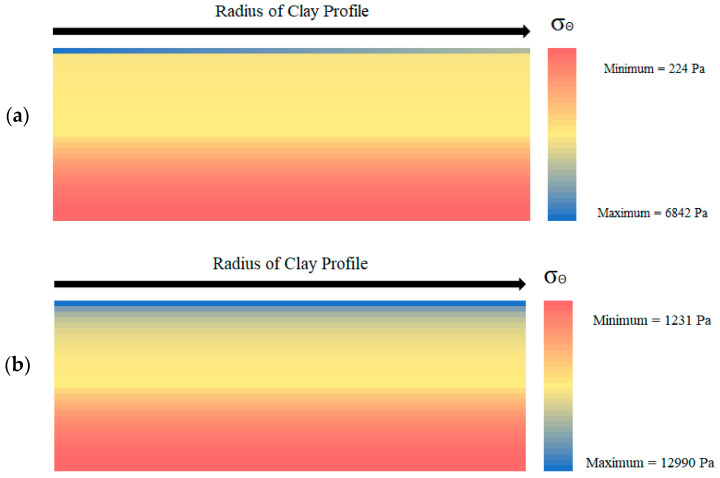
Hoop stress across the soil profile at the time of crack initiation for the (**a**) untreated and (**b**) xanthan-gum-treated soil.

In addition to the above stress plots, heat maps were generated of the clay profile at important times to observe the stress and displacement propagation throughout the whole clay layer, rather than just one point. Figure 13a displays the heat map for the hoop stress across the clay profile at the time of crack initiation for the untreated kaolin clay. The stresses vary as a function of depth up until the very top section of clay and then begin to decrease as a function of radius. This is because, as the stress formulation state switches from pre-wall crack to post-wall crack, the stress also switches from being predominantly a function of time to a function of radial distance. This trend of behavior continues for each of the fiber-aided soil models, albeit with increasing stress magnitudes. The stress profile at crack initiation for the xanthan gum model in Figure 13b displays a different pattern of stress propagation along with the soil layer in comparison with Figure 13a.

**Figure 14:**
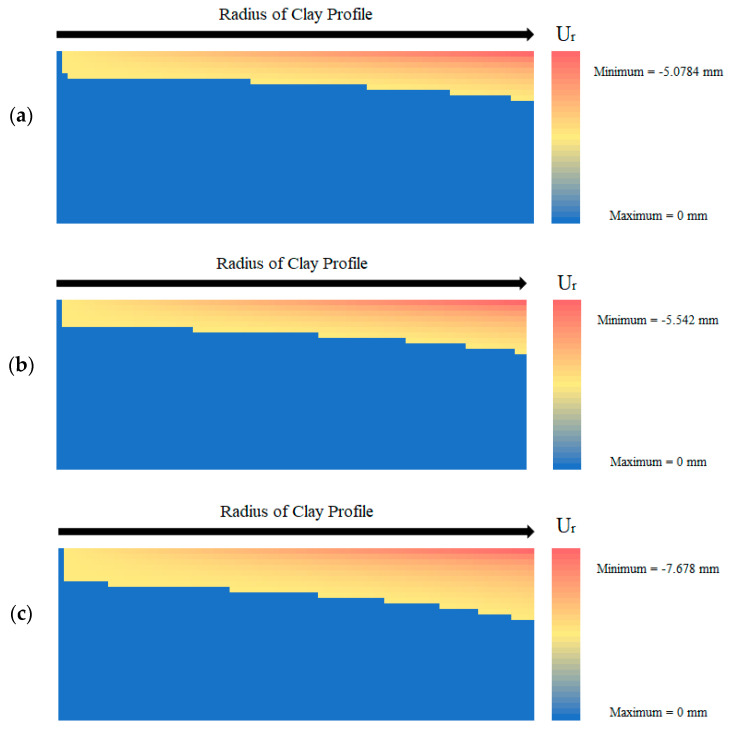

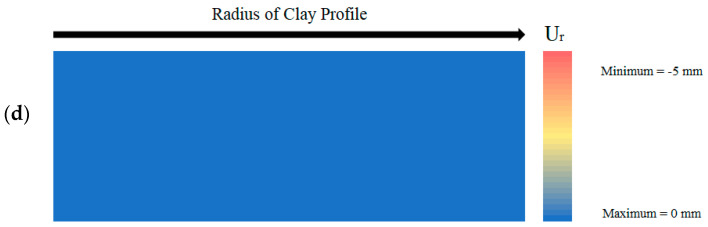
Displacement across the clay profile at the end of the analysis for the (**a**) untreated, (**b**) 0.5% fiber-treated, (**c**) 2% fiber-treated, and (**d**) xanthan-gum-treated soil.

On top of the stress propagation heat map, the radial displacement was also examined in this form (Figure 14). In Figure 14a, the radial displacement for the untreated sample at the last time step shows that the greatest radial movement (5.08 mm) occurs at the edges of the clay specimen, at the soil surface. In fact, the majority of the figure shows no displacement occurring. Additionally, no displacement occurs at the center of the profile as all shrinkage occurs in the negative radial direction, and the center should remain immobile. Figure 14b displays the displacement heat map for the 0.5% fiber content model. This simulation shows a slight increase in the overall radial displacement (5.54 mm) and shrinkage as well as the number of nodes where shrinkage occurs. Figure 14c maps the radial displacement within the soil layer for the 2.0% fiber content model. It shows the highest amount of shrinkage occurring in terms of magnitude with a maximum displacement of −7.678 mm in the radial direction. The fiber-reinforced soil model also possesses the highest number of nodes that experience movement. Finally, Figure 14d displays the displacement within the soil profile for the xanthan-gum-amended soil model. Because wall cracking never occurred, the radial displacement always remained zero, yielding the displacement heat map below.

**Figure 15:**
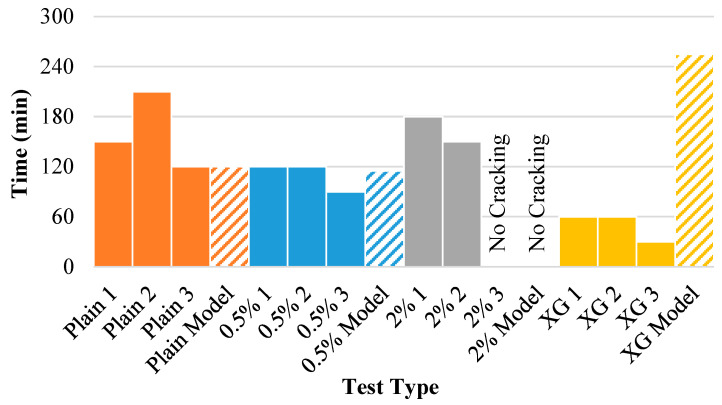
Internal crack time for each of the experiments and model results.

In addition, the crack initiation data returned from the model were compared to the experimental results. While the models generally predict the crack and wall crack to occur simultaneously, this was not always the case for the physical experiments. Additionally, while the model was able to be discretized into smaller time steps, the experimental results could only return values every 30 min. Figure 15 displays the internal crack time for each of the experiments (solid bar) as well as the model results (dashed bar). The model does reasonably well at predicting the crack time for the untreated soil and the 0.5% fiber-reinforced specimens. However, the gap between the model and experimental results is wider for the 2% fiber and the xanthan gum specimens. For the 2% fiber-reinforced specimens, when cracking did occur, the maximum width that occurred was 1.4 mm and the maximum length that occurred was 29.4 mm. In addition, the cracks showed little growth and were isolated, suggesting that they were more products of imperfections in the soil surface rather than soil failure.

**Figure 16:**
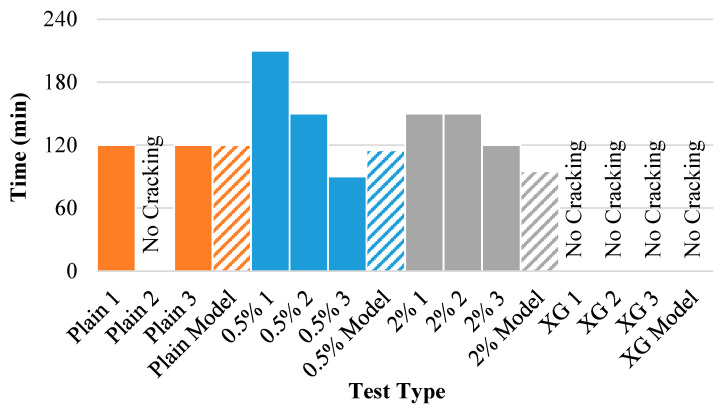
Wall crack time for each of the experiments and model results.

Figure 16 displays the wall crack time (time when the specimen separates from the mold wall) for each of the experiments (solid bars) as well as the model results (dashed bars). With the exception of outliers, the model approximates when the clay will separate from the mold wall. While one untreated soil specimen never detached from the mold, the model predicted the exact time at which the other two specimens would break away. In addition, the model predicts that the xanthan-gum-aided samples would never separate from the wall, which was the case for every tested specimen.

**Figure 17:**
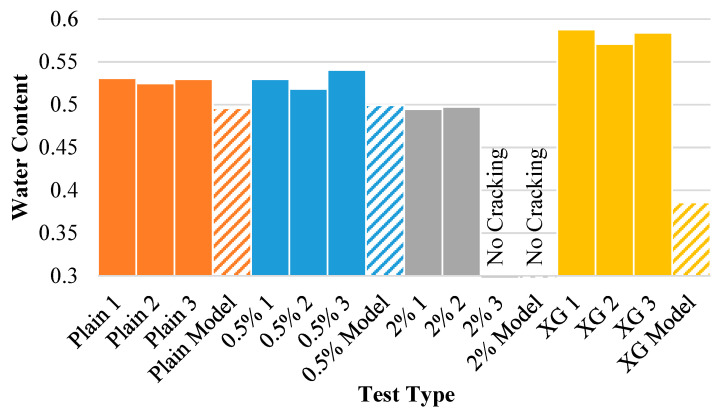
Internal crack water content for each of the experiments and model results.

Figure 17 displays the water content at crack initiation for each of the experiments as well as the model results. This figure shows that the model is even more accurate in this predictive measurement than predicting the time of crack initiation for the untreated soil and the 0.5% fiber-reinforced soil, though it slightly underestimates the time at which crack formation will occur. This is most likely because the model cannot take soil imperfections into account, which were found to expedite crack commencement in the physical experiments. Again, the model performs worse with the 2% fiber content specimens and the xanthan gum specimen. The reasoning for this deviation remains the same, however.

**Figure 18:**
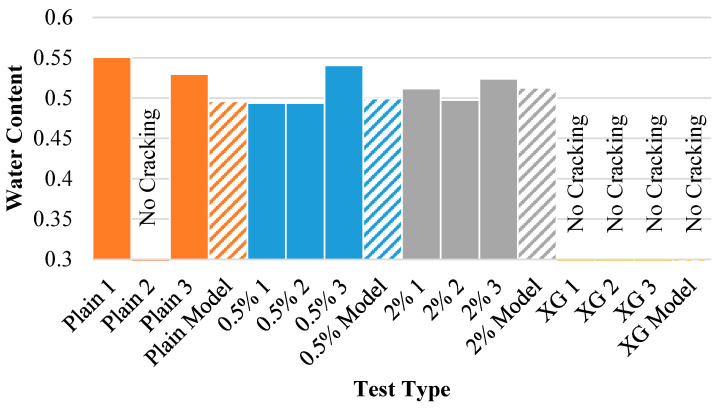
Water content at wall crack initiation for each of the experiments and model results.

Figure 18 displays the water content at the onset of the wall crack for each of the experiments (solid bars) as well as the model results (dashed bars). Once again, the model predicts the water content at which the clay will separate from the vertical mold wall, establishing a strong relationship between the experimental and mathematical data. Except for the untreated specimen that did not separate from the wall (Plain 2), the model provides a reasonable estimate for the water content at which this will occur. Going further, the 2% fiber content-aided soil provided a particularly strong prediction of water content at crack origination.

#### 4.2.3. Sensitivity Analysis

**Figure 19:**
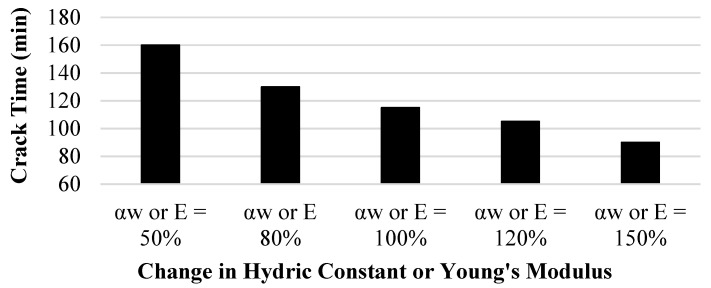
Hydric constant sensitivity analysis on crack initiation time.

A sensitivity analysis was performed on several of the material properties in order to determine their effect on the overall model behavior. The first parameter that was tested as part of the sensitivity analysis was the αw used as part of the model coupling process. Figure 19 shows that as the αw increases, the time of crack initiation decreases significantly. Young’s modulus has the same effect on the crack initiation time. This is because, in terms of stress, *E* only appears in Equations (25) and (26) or the pre-wall crack stress calculation, and because it shares the same relationship with each stress as the shrinkage strain.

**Figure 20:**
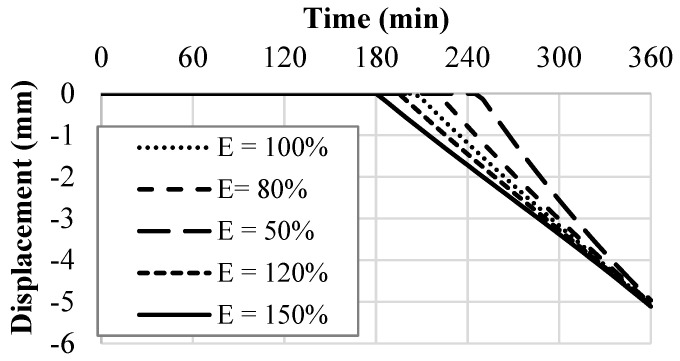
Young’s modulus sensitivity analysis on radial displacement.

Despite this, *E* also appears in Equation (29) or the radial displacement formula. Figure 20 shows how altering *E* affects the radial displacement as a function of time. As seen in Figure 20, an increase in *E* accelerates wall crack initiation, which leads to radial displacement occurring at an earlier time. However, Figure 20 shows that the rate of radial displacement decreases as *E* is increased. In fact, there appears to be a cross-over point near the end of the model’s duration, where the decreased *E* will begin to result in a larger displacement.

**Figure 21:**
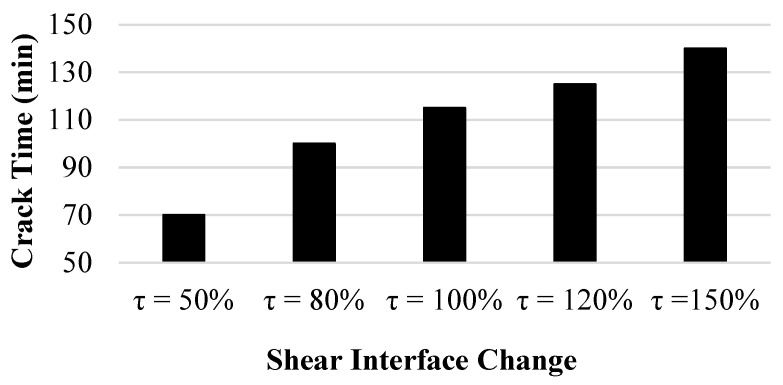
Interface shear strength sensitivity analysis on stress development.

The final parameter that was inspected as part of the sensitivity analysis was the interface shear strength (Figure 21). By decreasing the adhesion due to the decrease in the interface shear stress, clay separation from the mold occurred at an earlier time. The opposite occurred when increasing the adhesion due to an increase in the interface shear stress, as the wall crack happened at a later time. After the wall crack occurred, the changes in shear stress acted proportionally with the hoop stress, with the maximum hoop stress occurring with a 50% increase in shear stress and the minimum hoop stress accompanying the minimum interface shear stress.

## 5. Conclusions

The main objective of this study was to experimentally and numerically investigate eco-friendly soil improvement additives and their effect on the desiccation cracking behavior of soils. From the tests that were completed in this study, adding 2% fiber content was the most effective soil improvement technique. Even when cracks did occur, they were the smallest of the cracks that occurred in terms of both length and width. The cracks that formed in the specimens reinforced with 2% fiber content did not propagate or form large crack networks. Instead, crack growth leveled out and cracks were isolated incidents. This suggests that the cracking in these samples was a result of imperfections in the soil surface rather than stresses exceeding the unaltered tensile strength of the kaolin clay.

The xanthan-gum-aided samples were the most difficult to model as the behavior of the xanthan gum in the sample was not entirely a function of water content. Based on the results of the splitting tensile test on the completely dried xanthan gum specimens, the tensile strength should have been increased. However, these specimens cracked at a lower stress level than the untreated soil samples [50,51,52,53,54,55,56]. As a result, the hydro-mechanical model severely over-estimated the cracking time and water content for these samples. In addition, the cracks did not deepen as the water was removed from the soil. While the cracks in the other untreated specimens would eventually grow to reach the bottom of the mold, the cracks in the samples with xanthan gum would reach a certain depth and then stop. This behavior suggests that there is a time element to maximizing the strengthening behavior of the xanthan gum.

Following this line of thinking, xanthan gum has been shown to strengthen kaolin clay when completely dry and unrestrained, most notably in the unconfined compression test and splitting tensile test [23,24]. This suggests that the soil preparation for the xanthan-gum-aided specimens in the desiccation tests was not optimized. The benefits of xanthan gum were not harnessed in the controlled preparation environment that was necessary for a comparative analysis for soil improvement additives.

In terms of analyzing cracking behavior, the model was effective in explaining the crack pattern behavior for different soil improvement techniques. The radial direction of the cracks in the samples where wall cracking occurred can be explained by the hoop stress always exceeding the radial stress in that situation. As a result, cracking occurred in the direction perpendicular to the maximum stress, leading to the common crack pattern of cracks initiating centrally before radiating to the edge of the specimen. On the other hand, the samples treated with xanthan gum displayed a far more varied crack pattern, correlating to the stress conditions in the pre-wall crack stage, with the hoop stress and radial stress being equal in all lateral positions.

The model tended to underestimate the water content at crack initiation compared with the water content at crack initiation measured experimentally. For the untreated and 0.5% fiber content specimens, the underestimation was approximately 3–4%. This can be explained by the model predicting cracking to occur based solely on tensile strength versus stress, whereas natural cracking behavior is a function of soil surface conditions as well as stress conditions. The imperfect soil surface conditions could not be modeled. It is likely that the existence of soil surface imperfections leads to crack initiation occurring at greater water contents in the tested specimens.

## Data Availability

Some or all of the data, models, and code generated or used during the study are available from the corresponding author by request.
